# Cancer Surgery in the Elderly

**DOI:** 10.1100/2012/303852

**Published:** 2012-01-03

**Authors:** Gopal C. Kowdley, Nishant Merchant, James P. Richardson, Justin Somerville, Myriam Gorospe, Steven C. Cunningham

**Affiliations:** ^1^Department of Surgery, Saint Agnes Hospital Center, 900 Caton Avenue, Baltimore, MD 21229, USA; ^2^Department of Medicine, Saint Agnes Hospital Center, 900 Caton Avenue, Baltimore, MD 21229, USA; ^3^Laboratory of Molecular Biology and Immunology, National Institute on Aging, NIH, 251 Bayview Boulevard, Baltimore, MD 21224, USA

## Abstract

The proportions both of elderly patients in the world and of elderly patients with cancer are both increasing. In the evaluation of these patients, physiologic age, and not chronologic age, should be carefully considered in the decision-making process prior to both cancer screening and cancer treatment in an effort to avoid ageism. Many tools exist to help the practitioner determine the physiologic age of the patient, which allows for more appropriate and more individualized risk stratification, both in the pre- and postoperative periods as patients are evaluated for surgical treatments and monitored for surgical complications, respectively. During and after operations in the oncogeriatric populations, physiologic changes occuring that accompany aging include impaired stress response, increased senescence, and decreased immunity, all three of which impact the risk/benefit ratio associated with cancer surgery in the elderly.

## 1. Introduction

Due in large part to a steadily rising life expectancy in the most privileged parts of the world, currently greater than 80 years in much of Europe, North America, Australia, and New Zealand [1], cancer is an increasingly common lifetime event, occurring approximately 10-fold more frequently in the elderly than in younger patients [2–4]. The definition of “elderly” is rather elusive and less meaningful as physiologic age becomes recognized as more relevant than chronological age. Nevertheless, studies must use a cut-off value, such as 65 years [2, 3] and 85 years [4], to define the older population at higher risk of developing cancer. For those who survive to old age, the lifetime risk of developing any invasive cancer is higher for men (44%) than women (38%) [4].

Despite the growing emphasis on physiologic age versus chronologic age in the assessment of an individual's risk/benefit ratio when faced with the prospect of cancer surgery, there are several issues relevant to the elderly population (as identified by chronological age) as they face increasing rates of cancer. These issues include but are not limited to the underrepresentation of elderly patients in randomized clinical trials [5–7], the impact of social connections on risk in the elderly [8, 9], and the changing risk/benefit ratio associated with cancer screening, ageism among professional health providers, the possibility of increased risk of perioperative morbidity and mortality following cancer surgery, and the characterized physiologic changes associated with aging and impacting the ability of oncogeriatric patients to avoid cancer and to recover from cancer surgery. 

In the present paper, we will first discuss several salient points specific to the surgical care of the elderly patient, including fundamental consideration of the concept of chronologic versus physiologic age and how to best assess it, then moving to the presurgical issue of cancer screening in the elderly, then, once a diagnosis of cancer is established, to the issue of ageism in the decision-making process regarding cancer surgery in the elderly, and finally, once an operation is undertaken, some outcomes following cancer operations in the elderly and some age-specific mechanisms of immunity and carcinogenesis relevant to the oncogeriatric population.

## 2. Chronological Age versus Physiological Age

Ageism, or prejudice based on age alone, may be present when older patients come for evaluation of a cancer and are evaluated for their diagnosis and offered treatment recommendations. As one ages, a progressive but largely variable loss of physiological and cognitive function occurs and is relevant to the evaluation of an elderly cancer patient [10–13]. Due to this loss of function, along with increasing comorbidities, a more comprehensive assessment of the geriatric patient may be warranted. 

In the general home-dwelling geriatric population, an aggressive program of in-home comprehensive geriatric assessments (CGAs) may delay the development of disability and reduce permanent nursing home stays [14]. Similarly, in the oncogeriatric population, a CGA may help with clinical decision making [10–13, 15–17]. The scale is an assessment of not only an individual's general and oncologic health, as typically assessed in general medical and oncologic evaluations, respectively, but also includes a comprehensive assessment of functional status, nutrition, cognition, socioeconomic, and emotional conditions [13, 16] and has been found, even in a population of patients with good performance status, to add meaningfully to the assessment of oncogeriatric patients [18] who may be candidates for cancer surgery. Extermann and Hurria reviewed studies of CGA in older adults and concluded that CGA predicted survival, chemotherapy toxicity, postoperative morbidity, and mortality in cancer patients [17]. Although some have called for the use of CGA for all elderly patients, regardless of age, to guide oncologic treatment plans [19, 20], a survey of breast surgeons revealed that >80% of patients did not undergo a geriatric assessment routinely; rather, operative risk was based predominantly on the American Society of Anesthesiologists' (ASA') grade in 45% of respondents [21]. As the CGA is often time consuming, abbreviated screening scales to identify patients who would most benefit from a full CGA have been proposed as well [22, 23]. 

The preoperative assessment of cancer in the elderly (PACE) is another measure that has been used in geriatric oncology patients potentially undergoing operation. It began as a prospective, multi-institutional, international investigation aimed at defining the perioperative risk of oncogeriatric surgical candidates [24] and has subsequently been studied in multi-institutional investigations of oncogeriatric surgical patients [10, 11, 25, 26]. PACE extends CGA to include Satariano's index of comorbidities (SICs), mini-mental state inventory (MMS), activities of daily living (ADL), instrumental activities of daily living (IADL), geriatric depression scale (GDS), brief fatigue inventory (BFI), the Eastern Cooperative Oncology Group performance status (ECOG-PS), and the ASA (Table 1), in order to more specifically address operative risk faced by oncogeriatric patients. The goal was to measure short-term outcomes such as length of hospital stay and 30-day morbidity and mortality. PACE was shown to be effective in the preoperative assessment of elderly patients who underwent elective oncologic surgery: in a consecutive series of 460 elderly patients (≥70 years old), multivariate analysis identified moderate/severe fatigue, dependence in IADLs, and impaired PS as the most important independent predictors of postoperative complications [26]. Similarly, disability as assessed by dependence in ADLs and IADLs and impaired PS were associated with an extended hospital stay [26]. This and several other prospective studies support the use of formal geriatric assessments to improve outcomes in oncogeriatric patients (Table 2).

Acute-care admissions are associated with the development of age-related complications, such as delirium. In a population of 551 patients ≥65 years old and newly admitted to facilities following acute-care hospitalization, those who developed delirium were significantly older than those who did not [27]. In addition, patients with delirium had worse functional status than those without delirium as indicated by higher ADL and IADL scores [27]. Following operations, aging-related complications such as delirium are even more problematic than those in the nonoperative acute-care hospital setting and correlate independently with poor functional recovery. For example, in a series of 126 patients ≥65 years old undergoing hip replacement, delirium occurred in 52 (41%) patients and persisted in 20/52 (39%) at hospital discharge, 15/52 (32%) at 1 month, and 3/52 (6%) at 6 months [28]. Patients ≥80 years old, or with prefracture cognitive impairment, ADL impairment, and comorbidities, were more likely to develop delirium. However, even after adjustment for all of these factors, delirium was still associated with poor functional recovery at 1 month [28]. 

Several tools are available for preventing age-related complications such as delirium during the perioperative period. For example, given the common association between polypharmacy and associated adverse drug reactions in the oncogeriatric population, a formal medicine-review tool such as STOPP (screening tool of older persons' potentially inappropriate prescriptions) should be considered in all elderly patients undergoing cancer operations [29]. Also, regardless of the presence of polypharmacy, a preoperative geriatrics consultation has been shown by randomized controlled data to prevent delirium in patients following hip fracture [30].

Decisions regarding recommendations for cancer treatment in the elderly population will likely continue to be increasingly common in the upcoming years as the elderly population increases. Assessment tools such as those included in PACE provide an appropriate and useful means of avoiding ageism by basing recommendations on physiologic rather than solely on chronologic age. In this paper; therefore, the term “age” will often be used in a global sense, incorporating both chronologic age and physiologic age with the attendant comorbidities prevalent in the elderly population.

## 3. Cancer Screening

Cancer screening is the testing for an undiagnosed and asymptomatic cancer in individuals at reasonable risk for developing the cancer. In order to justify the risks of screening, the benefits of screening must outweigh the risks, which may be both direct, procedure-related risks and indirect result-related risks. Direct, procedure-related risks range from mild anxiety, pain, and discomfort associated with phlebotomy to severe procedure-related morbidity and mortality such as endoscopic perforations of hollow viscera. Indirect, result-related risks include both false-positive results that lead to unnecessary further diagnostic or treatment procedures and false-negative results that lead to delay in diagnosis. To optimize this risk/benefit ratio, certain characteristics of both the cancer and the screening test should be present (reviewed in [43]). An ideal screening test is safe, inexpensive, able to detect the cancer at an early stage, and able to do so accurately (with high sensitivity and specificity). An ideal cancer for screening is one that, if missed, levies a high cost of morbidity and mortality as well as financial cost to society during its treatment, one whose incidence and prevalence are high enough to justify screening, one with a well-characterized natural history and biology, and one for which effective treatment options exist (Table 2). In the very elderly individual, who is at increasing risk of death due to cardiovascular, cerebrovascular, and other noncancer diseases, the question of when—if ever—to stop cancer screening becomes increasingly relevant.

### 3.1. Breast Cancer Screening

Screening for breast cancer is commonly accepted for female patients beginning at age 40, and the American Cancer Society (ACS) recommends continuing mammographic screening as long as a woman is in good health [1]. In women aged 40–74, mammography has been shown in a 2002's review of randomized controlled trials (RCTs) for the US Preventive Services Task Force (USPSTF) to reduce breast cancer mortality rates [44]. There is a lack of good evidence, however, for similar recommendations in patients older than 74 years. For these patients >74 years of age, the USPSTF study concluded that there is insufficient data to make recommendations in this age group [45], and some have indeed argued against screening mammography in women >80 years old, citing concerns that screening may be more harmful than beneficial [46]. 

Given the insufficient data in real people, modeled people have been studied in a stochastic model using the Monte-Carlo simulation, in which the natural history of breast cancer is used to evaluate costs, harms, and benefits associated with screening cessation at various ages [47]. Based on age-dependent biology and incorporating a cost analysis, it was found that the benefits of mammography beyond the age of 79 were too low relative to the costs to justify their continued biennial use [47]. Compared to stopping screening at age 70, extended screening to age 79 saved only 2.4 days of life per woman for the entire population but provided an additional 24.9 days of life per woman destined to develop breast cancer at an incremental cost of $82,063 per life year saved [47]. The authors concluded that extending screening beyond age 79 may be reasonable for women in the top quartile of life expectancies [47]. 

Other ways to make the continued use of mammograms cost effective in this group of very elderly women include expanding screening intervals or selectively screening only patients with an increased risk of developing breast cancer or at decreased risk of natural-cause death in very healthy individuals. Indeed, there is evidence that physicians and patients are already making screening decisions based upon patients expected mortality in this age group: in a series of 5554 Medicare beneficiaries, use of screening mammography in older women ≥65 years old was significantly associated with their decreased 4-year risk of mortality rather than with age alone, suggesting that, when making decisions regarding screening in elderly women, patients and providers do already appropriately consider general health, prognosis, and life expectancy [48].

### 3.2. Prostate Cancer Screening

Similar to breast cancer screening, prostate cancer screening is controversial in the elderly with conflicting data published [49]. The ACS recommends that screening cease once men reach a life expectancy of <10 years, since such men “are not likely to benefit,” but appropriately caution that “[o]verall health status, and not age alone, is important when making decisions about screening” [50]. Whereas a study of over 2000 elderly men from the Duke Prostate Cancer Database concluded that it may be safe to discontinue screening in men 75–80 years old [51], a recent query of the Center for Prostate Disease Research database has shown that men >70 years old had a statistically significant higher prediagnosis prostate specific antigen increase, higher clinical stage, and higher biopsy grade, compared with their younger counterparts, raising concern regarding cessation of screening in this age group [52]. As with breast cancer recommendations, the soundest course of action regarding prostate cancer screening decisions is likely to consider patients on a case-by-case basis, taking into account prognosis and overall health status, as supported by both consensus statements [53] and by the ACS recommendation [50] to consider overall health status as opposed to age alone when making decisions about screening for prostate cancer in the very elderly.

### 3.3. Colorectal Cancer Screening

Given its accessibility to screening and its high incidence of cancer, the colon, like the breast and the prostate, is highly amenable to screening such that colorectal cancer (CRC) is a largely preventable disease, the incidence and mortality of which have decreased in association with increased screening. However, as with prostate and breast cancer, there may come an age when screening may no longer provide a favorable risk/benefit ratio. The USPSTF currently recommends against routine screening for CRC in the elderly population 76 to 85 years of age, with the grade C recommended caveat such that, for individual patients in this group, screening may be considered [54]. A grade D recommendation is provided by the USPSTF against screening for CRC in those >85 years old [54]. As evidenced; however, by the low grades of the recommendations, little evidence exists to guide decision making, consistent with a recent systematic review of the scant published evidence [55]. 

In the absence of convincing evidence, surveyed physicians have appropriately considered a wide range of factors, including not only clinical factors such as age, life expectancy, comorbidities, and functional status but also psychosocial factors, such as personality, previous screening behavior, social support, and the physician-patient relationship [56]. In an effort to improve decision making and with the understanding that patient preferences matter in such cases of scant data, Lewis et al. used formative research and cognitive testing to develop and refine a decision aid designed to promote individualized decision making regarding CRC screening [57]. In this uncontrolled trial enrolling 46 patients ≥75 years of age, use of the decision aid was associated with a logarithmic increase in the proportion of participants with adequate knowledge enabling them to participate in a meaningful way in decision making (from 4% to 41%), suggesting that with adequate education elderly patients can and should be able to participate in the decision to choose the risks of screening versus the risks of missing an undiagnosed CRC [57]. Given that risks of screening may be greater in the elderly than in middle-aged individuals [58, 59], the decision to proceed with screening in the elderly must be well informed and made with caution.

In general, risk of colorectal (or breast or prostate) cancer should be assessed with a complete history (personal or familial history of cancer, history or prior radiation, etc.) and a complete annual physical exam, including digital rectal exam, to assess overall health and estimate life expectancy, all of which entail minimal to no risk to the geriatric patient. Screening for patients with a life expectancy of >5 years should be considered. With colorectal cancer in particular, it is important to note that many screening tools exist, each with its own risk profile, including annual fecal occult blood testing alone, flexible sigmoidoscopy every 5 years with or without fecal occult blood testing, barium enema every 5 years, or colonoscopy every 10 years.

## 4. Ageism in Decision-Making Process regarding Cancer Surgery in the Elderly?

Once the diagnosis of cancer has been made in an elderly individual, whether by screening or by investigation of symptoms, treatment decisions have been shown, for better or for worse, to correlate with chronologic age. In a large survey of primary care providers in France, for instance, in a multivariate analysis, chronological age of the patient was highly associated with the decision not to refer patients with advanced (not defined) cancer to oncologic specialties (odds ratio 0.55; 95% confidence interval 0.35–0.86; *P* = 0.009) [60]. 

If elderly patients are referred to an oncologic specialty, further ageism may exist there too: in a survey of 1408 French medical and radiation oncologists to whom breast cancer patients were referred, significant differences in treatment choice were observed depending only on patient age [61]. Following the 1990's National Institutes of Health Consensus Conference recommendation that patients with stage III CRC receive adjuvant chemotherapy to increase survival [62], prospective data from 85,934 such patients were entered into the National Cancer Data Base and studied to determine whether adjuvant therapy failed to benefit any specific sets of patients; although elderly patients derived the same benefit as younger patients, they were less frequently treated [63].

Underscoring the importance of the above-discussed CGA in elderly cancer patients, a study of 161 such patients who were referred for geriatric consultation, and who underwent CGA, found that the CGA significantly influenced the final cancer-related treatment decisions in 82% of the elderly cancer patients [64]. Similarly, tools exist within surgical subspecialties to assist in the risk stratification of oncogeriatric patients being considered for curative-intent cancer surgery [65].

## 5. Studies of Cancer Surgery in Elderly Patients

Once the decision has been made to proceed with curative-intent operation in an elderly patient, do operative outcomes support the decision to have elderly patients subjected to invasive procedures? Given that the greatest risks are associated with the largest operations [66], review of major thoracic and abdominal and operations will prove most useful to answer this question. 

In the thoracic surgical oncology literature, some large population-based studies, such as that by Owonikoko et al. analyzing the Surveillance, Epidemiology, and End Results (SEER) Database, including over 45,000 patients, have suggested that patients >80 years old were less likely to undergo operation or radiation and had inferior outcomes when compared with younger patients [67]. However, analysis of more recent studies focusing on age as an independent risk factor support the decreasing importance of chronologic age alone in the preoperative risk evaluation of patients prior to esophageal [68, 69] and pulmonary [70–73] resection.

Similarly, although large population-based studies in the pancreatic literature suggest worse short-term outcomes in older, compared to younger, patients [74, 75], it is likely that “age” in these population-based studies was simply a surrogate for chronic illness. When large series of elderly patients undergoing major pancreatic and hepatobiliary operations are analyzed, chronological age turns out not to be a meaningful risk factor, although all agree that physiologic age as described above is essential to consider [76–80]. When the contribution of chronologic age was isolated statistically using logistic regression modeling with pseudo *r* ^2^ analysis in one of the world's largest series of pancreaticoduodenectomy, age alone was found to contribute less than 1% to morbidity and mortality, whereas chronic obstructive pulmonary disease and coronary artery disease had a nearly 4-fold and 5-fold increased impact, respectively, [76]. 

In addition to physiologic health; however, patient goals must be taken into account when considering a major pancreatic resection, especially one not for known cancer, but for a premalignant lesion such as branch-duct intraductal papillary mucinous neoplasm (BD IPMN), for which consensus guidelines recommend resection at the 3-cm threshold in the general population [81, 82]. As with considerations of colorectal screening discussed above [56], the personal goals and preferences of the elderly patient are highly relevant and warrant careful consideration. To address this issue, Weinberg et al. employed the Markov modeling of the competing goals of maximizing survival and quality of life [83]. The decision to resect or to surveil a BD IPMN depended on patient age and comorbidities, on the size of the cyst, and whether the modeled patient placed more valued on quality or quantity of life: those valuings primarily survival, irrespective of quality of life, were found to benefit most from resection of lesions >2 cm, whereas patients valuing quality of life over longevity derived a greater benefit from a 3-cm threshold for resection.

Leaving aside the question of differences in the outcomes of morbidity and mortality in older versus younger patients, there are clearly other differences. For example, differences in histology exist in the elderly: an analysis of elderly lung cancer patients in the SEER database revealed fewer cases of adenocarcinoma in older patients: 33% in those <70 years old compared with 27% in those aged 70 to 79 years and 23% in patients ≥80 years old [67]. Even more relevant to the surgical treatment of elderly cancer patients is a study of the National Cancer Data Base (NCBD), including 142,009 N0M0 patients who underwent colectomy for adenocarcinoma: adequate (≥12 nodes harvested) lymph node counts obtained in only 41% if patients >78 years old (median 10 nodes) compared to 48% in patients <67 years old (median 11 nodes) (*P* < 0.0001 for the percent of patients with adequate nodal harvests, but NS for the median nodes harvested). Given that one of the key prognostic indicators of colorectal cancer is the number of lymph nodes harvested during surgical resection, this study highlights the need for increased use of techniques to increase the harvest in elderly patients. Increased awareness of such age-related differences is mandatory for those providing surgical treatment for the increasing elderly population of cancer patients.

## 6. Age-Specific Mechanisms of Carcinogenesis and Immunity

Given that physiologic age is so much more appropriate than chronological age in the evaluation of the oncogeriatric patient, what is it that makes a physiologically old patient old? Are these some of the same mechanisms that contribute to the increased incidence of cancer in the elderly?

### 6.1. Impaired Stress Response in the Elderly (Impaired Repair)

The functions of cells change profoundly with advancing age. Among the best-documented changes is the ability of cells to respond to proliferative and stress-causing agents [84, 85]. The cells of a young person respond optimally to such agents, mounting an appropriate molecular response that preserves tissue homeostasis and maintains health. By contrast, the cells of an elderly individual responding to the same stimuli mount aberrant molecular responses which can be inadequate in magnitude, quality, and/or timing [86]. In turn, this impairment underlies much of the age-related physiologic decline, like the loss of muscle mass, immune function, skin elasticity, and fertility, as well and the increase in age-associated pathologies like neurodegeneration, cardiovascular disease, diabetes, and cancer [87–92].

### 6.2. Increased Senescence (an Anticancer Mechanism) Can Be Procarcinogenic in the Elderly

A person's cells frequently encounter damage from internal and external sources. To preserve tissue function, mild damage is typically repaired, whereas severe damage may trigger cell death via apoptosis or necrosis. However, some cellular damage can be oncogenic, for example, DNA mutations that activate oncogenes or inactivate tumor suppressor genes (reviewed in [93]). Such promalignant mutations typically trigger a process named cellular senescence, controlled by the p53 and RB tumor suppressor proteins, whereby cells cease cell division but remain metabolically active. In young persons, cellular senescence is a potent tumor-suppressive mechanism and promotes early-life survival; in the elderly, however, cellular senescence can also limit longevity by contributing to aging and to age-related diseases, including cancer [94]. In this regard, senescent cells can promote cancer by stimulating the so-called senescence-associated secretory phenotype (SASP), whereby cells secrete high levels of cytokines and chemokines, triggering a strong proinflammatory response. A proinflammatory state can be pro-oncogenic by, for example, favoring the proliferation, migration, and invasion of premalignant cells. However, the picture is more complex, since SASP can also maintain the senescent phenotype and, thus, prevent oncogenic progression [95].

### 6.3. Decreased Immune Function in the Elderly

The immune system plays a key role not only in the prevention and management of postoperative complications following cancer surgery but also in the defense of the body against cancer development and progression. Cancer immunosurveillance is the ability of the immune system to prevent tumorigenesis and the spread of cancer cells by recognizing cancer antigens and preventing cancer cell proliferation. If the immunosurveillance is efficient, cancerous cells are inhibited or eliminated, thus, reducing or preventing cancer growth [96]. With advancing age, however, several well-known alterations impair both the innate immunity (immunosurveillance) and the adaptive immunity (type and amount of T and B lymphocytes, as well as the cytokines they produce) [97]. These detrimental changes are collectively termed *immunosenescence* [98, 99]. Although definitive evidence is needed, this decline in immune strength is widely considered to contribute to the higher incidence of cancer among the elderly.

## Figures and Tables

**Table 1:** Preoperative assessment of cancer in the elderly (PACE): a comprehensive assessment of underlying characteristics of elderly cancer patients prior to elective surgery.

First author	Name of test	Abbreviation	[Ref]
Satariano	Satariano's modified index of comorbidities	SIC	[31]
Folstein	Mini-mental state	MMS	[32]
Katz	Activities of daily living	ADL	[33]
Lawton	Instrumental activities of daily living	IADL	[34]
Yesavage	Geriatric depression scale	GDS	[35]
Mendoza	Brief fatigue inventory	BFI	[36]
Oken	ECOG performance status	PS	[37]
ASA	American Society of Anesthesiologists' grade	ASA	[38, 39]

**Table 2:** Prospective studies on the effect of geriatric assessment before cancer surgery.

First author, year [ref]	*N*	Age (min, mean)	Cancer	Intervention	Results
McCorkle et al., 2000 [40]	375	60, NR	Solid	Advance-practice nurses (APNs) for postsurgical home care	Improved survival with APNs
Repetto et al., 2002 [18]	363	65, 73	Solid and hematologic	CGA	CGA added to PS, even PS is high
Freyer et al., 2005 [41]	83	70, 76	Ovarian	CGA to predict STox; patient autonomy; comorbidities; nutritional status	CGA predicted toxicity and overall survival
Pope et al. (PACE), 2006 [25]	460	65, 77	Breast, GI, GU	PACE	PACE associated with complications
Goodwin et al., 2003 [42]	335	65, ~72	Breast	Nurse case management	Improved management
